# Retraction Note: Development of a stemness-related prognostic index to provide therapeutic strategies for bladder cancer

**DOI:** 10.1038/s41698-026-01712-7

**Published:** 2026-10-07

**Authors:** Shi Fu, Zhiyong Tan, Hongjin Shi, Junhao Chen, Yawei Zhang, Chunming Guo, Wei Feng, Haole Xu, Jiansong Wang, Haifeng Wang

**Affiliations:** 1 Department of Urology, The Second Affiliated Hospital of Kunming Medical University, Kunming, China; 2 Yunnan Clinical Medical Center of Urological Disease, Kunming, China; 3 Kunming Medical University, Kunming, China; 4 School for Life Science, Yunnan University, Kunming, China

Retraction to: *npj Precision Oncology* https://doi.org/10.1038/s41698-024-00510-3, published online 20 January 2024

The Editor-in-Chief has retracted this article as the authors were unable to provide documentary evidence of ethical approval. The authors have not responded to any correspondence from the publisher about this retraction.

